# Nrf2 protects against pulmonary fibrosis by regulating the lung oxidant level and Th1/Th2 balance

**DOI:** 10.1186/1465-9921-11-31

**Published:** 2010-03-18

**Authors:** Norihiro Kikuchi, Yukio Ishii, Yuko Morishima, Yuichi Yageta, Norihiro Haraguchi, Ken Itoh, Masayuki Yamamoto, Nobuyuki Hizawa

**Affiliations:** 1 Department of Respiratory Medicine, Graduate School of Comprehensive Human Sciences, University of Tsukuba, Tsukuba, Japan; 2 Center for Advanced Medical Research, Hirosaki University School of Medicine, Hirosaki, Japan; 3 Department of Medical Biochemistry, Tohoku University Graduate School of Medicine, Sendai, Japan

## Abstract

**Background:**

Pulmonary fibrosis is a progressive and lethal disorder. Although the precise mechanisms of pulmonary fibrosis are not fully understood, oxidant/antioxidant and Th1/Th2 balances may play an important role in many of the processes of inflammation and fibrosis. The transcription factor Nrf2 acts as a critical regulator for various inflammatory and immune responses by controlling oxidative stress. We therefore investigated the protective role of Nrf2 against the development of pulmonary fibrosis.

**Methods:**

To generate pulmonary fibrosis, both wild-type C57BL/6 mice and Nrf2-deficient mice of the same background were administered bleomycin intratracheally.

**Results:**

The survival of Nrf2-deficient mice after bleomycin administration was significantly lower than that of wild-type mice. The degree of bleomycin-induced initial pulmonary inflammation and pulmonary fibrosis was much more severe in Nrf2-deficient mice than in wild-type mice. The expression of antioxidant enzymes and phase II detoxifying enzymes was significantly reduced in the lungs of Nrf2-deficient mice, concomitant with an elevation of lung 8-isoprostane level, compared with wild-type mice. The expression of Th2 cytokines, such as interleukin-4 and interleukin-13, was significantly elevated in the lungs of Nrf2-deficient mice with an increase in the number of Th2 cells that express GATA-binding protein 3.

**Conclusions:**

The results indicated that Nrf2 protects against the development of pulmonary fibrosis by regulating the cellular redox level and lung Th1/Th2 balance. Thus, Nrf2 might be an important genetic factor in the determination of susceptibility to pulmonary fibrosis.

## Introduction

Pulmonary fibrosis is a chronic progressive disorder in which excessive deposition of extracellular matrix as a result of tissue injury leads to irreversible scarring of interstitial lung tissue [1,2]. Although the precise pathologic mechanisms of pulmonary fibrosis are not fully understood, fibrosis is thought to be the result of an aberrant wound-healing response to sequential lung injury [3].

Oxidative stress may play an important role in this process because excessive levels of reactive oxygen species (ROS) may damage cellular macromolecules such as DNAs, lipids, and proteins, leading to oxidative stress-induced tissue injury [4,5]. To limit the potential toxicity of ROS, animals possess several cellular and extracellular enzymatic and small molecular antioxidant systems. Among these, classic antioxidant enzymes, including superoxide dismutases, catalase, and glutathione peroxidases, directly inactivate ROS and prevent ROS-initiated reactions [6]. Phase II detoxifying enzymes, including glutathione-S-transferase (GSTs), NADP(H): quinone oxidoreductase (NQO1), and glutamate-cysteine ligase catalytic (GCLC), also indirectly act as antioxidant enzymes by controlling biosynthesis/recycling of thiols or facilitating excretion of oxidized reactive secondary metabolites. In addition, stress-response proteins such as heme oxygenase-1 (HO-1) and peroxiredoxin I (Prx-I) are cytoprotective against various oxidant or prooxidant insults [7,8]. The decreased glutathione levels observed in the bronchoalveolar lavage (BAL) fluids and sputum of patients with idiopathic pulmonary fibrosis suggested that antioxidant capacity is decreased in the lungs of patients with this disease [9].

There are several factors that modify wound healing and the degree of fibrosis. Among them, an inflammatory phenotype (Th1 or Th2) is thought to be important as a host factor to modulate tissue injury and fibrosis. In idiopathic pulmonary fibrosis, the inflammatory response closely resembles a Th2-type immune response, with increases in the number of eosinophils and mast cells, and increased amounts of Th2 cytokines such as interleukin (IL)-4 and IL-13 [10,11]. In murine models, animals whose response to tissue injury is predominantly of the Th2 type are more prone to pulmonary fibrosis after lung injury than those with a predominantly Th1 response [12].

Nrf2 is a member of the family of cap'n'collar basic leucine zipper transcription factors and has been identified as a pivotal factor in the coordinated induction of antioxidant and phase II detoxifying enzymes under the regulatory influence of the antioxidant response element (ARE) [13,14]. Indeed, Nrf2 plays essential roles in protection against oxidant-induced pulmonary inflammation and fibrosis [15-18]. Recent studies have demonstrated that Nrf2 also modifies Th1/Th2 skewing by regulating oxidative stress [19,20]. It is therefore likely that Nrf2 is a critical host factor in the determination of individual susceptibility to pulmonary fibrosis. In the present study, we investigated the protective role of Nrf2 against the development of bleomycin-induced pulmonary inflammation and fibrosis at the cellular and molecular levels using mice lacking Nrf2.

## Methods

### Animals and exposure to bleomycin

Nrf2-deficient (*Nrf2-/-*) mice with an ICR/129sv background were backcrossed with C57BL/6 mice for eight generations. C57BL/6 WT mice were purchased from Charles River Japan (Kanagawa, Japan). All the mice used in this study were 6 to 8 weeks old and maintained in our animal facilities under specific pathogen-free conditions. All animal studies were approved by the Institutional Review Board. Mice were administered bleomycin (5 mg/kg; Calbiochem, San Diego, CA) or saline intratracheally.

### Histopathologic assessment

The lungs were removed 1, 3, and 28 days after bleomycin or saline administration. Following fixation, the lungs were embedded in paraffin. The sections were then stained with Masson's trichrome stain. The grade of pulmonary fibrosis was scored on a scale of 0 to 8 using a previously described scoring method [21]. After the examination of 30 randomly chosen regions in each sample at a magnification of × 100, the mean score of all the fields was taken as the fibrosis score in each sample.

### Assessment of collagen synthesis

Collagen synthesis was assessed using a hydroxyproline assay. The mice were anesthetized, and the lungs were removed 28 days after bleomycin or saline administration. Hydroxyproline content was measured as reported previously [22].

### Bronchoalveolar lavage (BAL)

The lungs of terminally anesthetized mice were lavaged with six sequential 1 ml aliquots of PBS 1, 3, and 8 days after bleomycin administration. Following centrifugation, the supernatant of the first BAL was used for analysis of lactate dehydrogenase (LDH) activity using standard NADH-linked enzymatic assay procedures as previously described [23]. Cells were counted using a hemocytometer and a differential cell count was performed by standard light microscopy based on staining with Diff-Quik (American Scientific Product, Obetz, OH).

### Measurement of cytokines

The concentrations of tumor necrosis factor (TNF)-α and macrophage inflammatory protein-2 (MIP-2) in the supernatant of the first BAL fluid were determined by enzyme-linked immunosorbent assay (ELISA) according to the manufacturer's instructions (TNF-α : BioSource International, Camarillo, CA; MIP-2: R&D systems, Minneapolis, MN). The concentrations of IL-4, IL-13, and interferon-γ (IFN-γ) in the lung homogenates were also determined by ELISA (R&D systems).

### Reverse transcription-polymerase chain reaction (RT-PCR)

Seven days after bleomycin or saline administration, total RNA was extracted from lung tissues, and real-time quantitative RT-PCR was performed using an ABI7700 sequence detector (Applied Biosystems, Foster City, CA). The ready-made PCR primers of GST-P1, GCLS, NQO1, Prx-I, HO-1, T-bet, and GATA-binding protein 3 (GATA3; Applied Biosystems) were used in this study. The results were normalized by GAPDH gene expression.

### Measurement of 8-isoprostane

The concentration of 8-isoprostane in the lung homogenates was determined by ELISA (Cayman Chemicals, Ann Arbor, MI).

### Flow cytometry

Seven days after bleomycin or saline administration, the lungs were removed, minced, and incubated with RPMI 1640 containing 10% fetal bovine serum and 75 U/ml collagenase (type 1; Sigma Chemical Co., St. Louis, MO) at 37°C for 90 minutes. The cells were then filtered through 20-μm nylon mesh. The cell suspensions were stained with anti-CD4, anti-CD8, anti-CXCR3, and anti-CCR3 antibodies (BD PharMingen, San Diego, CA), respectively. After staining, the cells were analyzed by flow cytometry using a FACSCaliber flow cytometer with CellQuest software (BD Biosciences, San Jose, CA).

Levels of IFN-γ and IL-4 production in T cells were determined by flow cytometric intracellular cytokine analysis as previously described [24]. Briefly, the cells were resuspended in RPMI 1640 containing 10% FCS, incubated with PMA (50 ng/ml; Sigma) and ionomycin (500 ng/ml; Sigma) for 2 h, and then incubated with brefeldin A (10 μg/ml; Sigma) for 2 h at 37°C. The cells were stained with PE-conjugated anti-mouse IFN-γ or with APC-conjugated anti-mouse IL-4 antibodies (BD PharMingen) and fixed with 2% paraformaldehyde-PBS solution.

### Western blotting

One day after bleomycin or saline administration, the lungs were removed and nuclear fractions were prepared using a Nuclear Extraction Kit (Panomics, Redwood City, CA) according to the manufacturer's protocol. The nuclear proteins were separated on 5-15% gradient SDS-polyacrylamide gels and transferred onto a polyvinylidene difluoride membrane. The membrane was stained immunochemically using an antibody against nuclear factor-κB (NF-kB) p65 subunit (Santa Cruz Biotechnology, Inc., Santa Cruz, CA). Immunoreactive bands were detected using ECL Western blotting detection reagents (Amersham, Buckinghamshire, UK). Lamin B was used as an internal control.

### Statistics

Data were expressed as the mean ± SEM. Comparisons of data among the experimental groups were performed using ANOVA and Scheffe's test. The survival curves were analyzed using the log-rank test. Values of *p *< 0.05 were considered to be statistically significant.

## Results

### Nrf2-/- mice are highly susceptible to bleomycin

**Figure 1:**
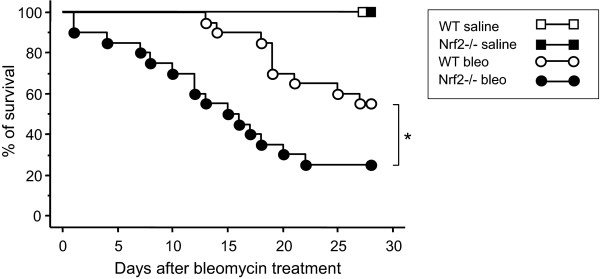
***Nrf2-/- *mice are highly susceptible to bleomycin**. Survival of WT mice and *Nrf2-/- *mice after treatment with bleomycin (bleo) or saline. n = 20 in each group. *Significant difference between WT mice and Nrf2-/- mice (p < 0.05).

We first evaluated the survival of mice after bleomycin administration. Forty-five percent of the WT C57BL/6 mice and seventy-five percent of the *Nrf2-/- *mice died within 28 days after bleomycin administration (Figure 1). The survival rate after bleomycin treatment was significantly decreased in *Nrf2-/- *mice compared with WT mice. No mice died in the saline-administered control group of either genotype (Figure 1).

### Bleomycin-induced initial pulmonary inflammation is enhancedin Nrf2-/- mice

**Figure 2:**
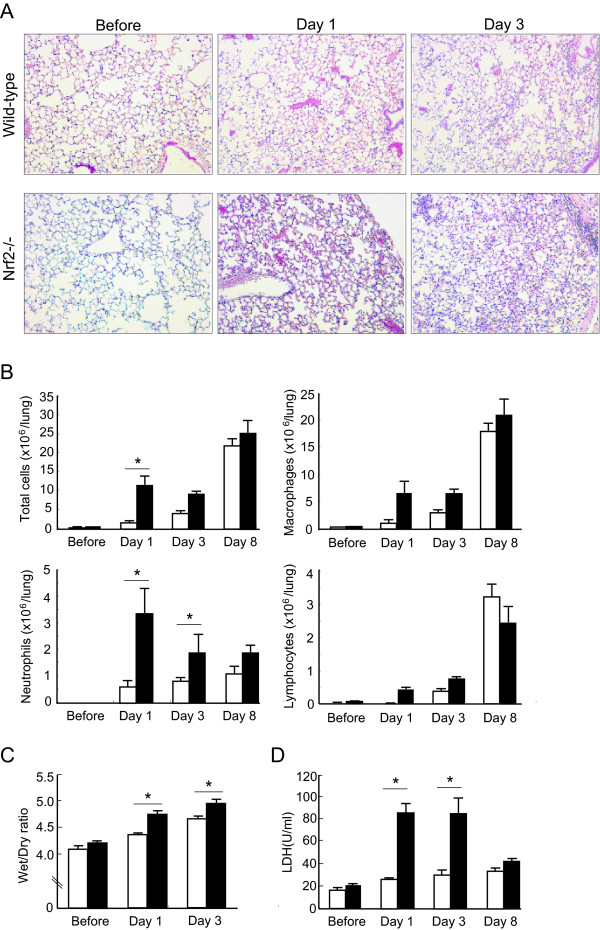
**Bleomycin-induced pulmonary inflammation is enhanced in *Nrf2-/- *mice**. **(A) **Representative photographs of lungs from WT mice and *Nrf2-/- *mice before, and 1 day (Day 1), and 3 days (Day 3) after treatment with bleomycin. Magnification: ×40. **(B) **The numbers of total cells, macrophages, neutrophils, and lymphocytes in bronchoalveolar lavage fluid of WT mice (open bars) and *Nrf2-/- *mice (solid bars) before, and 1 day (Day 1), 3 days (Day 3), and 8 days (Day 8) after treatment with bleomycin. n = 8 in each group. **(C) **Lung wet-to-dry ratio of WT mice (open bars) and *Nrf2-/- *mice (solid bars) before, and 1 day (Day 1), and 3 days (Day 3) after treatment with bleomycin. n = 8 in each group. **(D) **The activity of lactate dehydrogenase (LDH) in bronchoalveolar lavage fluid of WT mice (open bars) and *Nrf2-/- *mice (solid bars) before, and 1 day (Day 1), 3 days (Day 3), and 8 days (Day 8) after treatment with bleomycin. n = 8 in each group. *Significant difference (*p*<0.05) between WT mice and *Nrf2-/- *mice.

We assessed the degree of bleomycin-induced acute lung inflammation in both WT mice and *Nrf2-/- *mice. At the light microscopic level, inflammatory cell infiltration into the airspaces and alveolar septal edema, which are characteristic pathological findings of acute lung injury, were observed in the lungs of both genotypes of mice 1 day and 3 days after bleomycin administration (Figure 2A, Day 1 and Day 3). However, the degree of acute lung injury was much more severe in the lungs of *Nrf2-/- *mice, compared with WT mice, at both 1 day and 3 days (Figure 2A, Day 1 and Day 3). No pathological abnormalities were observed in the lungs of either genotype before bleomycin administration (Figure 2A, Before).

We next assessed the number of inflammatory cells in BAL fluid. The number of total cells recovered by BAL increased after bleomycin administration in both genotypes of mice. Between the genotypes, the number of total cells in BAL fluid was significantly higher in *Nrf2-/- *mice than in WT mice 1 day after bleomycin administration (Figure 2B, Total cells). The number of lavageable neutrophils was significantly higher in *Nrf2-/- *mice than in WT mice at 1 and 3 days after bleomycin administration (Figure 2B, Neutrophils). Although the numbers of macrophages and lymphocytes in BAL fluid were increased in both genotypes after bleomycin administration, the numbers were not significantly different between the genotypes (Figures 2B, Macrophages and Lymphocytes).

We next assessed the lung wet-dry ratio and LDH activity in BAL fluid as indicators of lung permeability damage and lung cell damage, respectively. The lung wet-dry ratio was significantly higher in *Nrf2-/- *mice than in WT mice at both 1 and 3 days after bleomycin administration (Figure 2C). LDH activity in BAL fluid was also significantly higher in *Nrf2-/- *mice than in WT mice at both 1 and 3 days after bleomycin administration (Figure 2D). These findings suggest that mice lacking Nrf2 are more susceptible to bleomycin-induced acute pulmonary inflammation.

### Bleomycin-induced pulmonary fibrosis is enhanced in Nrf2-/-mice

**Figure 3:**
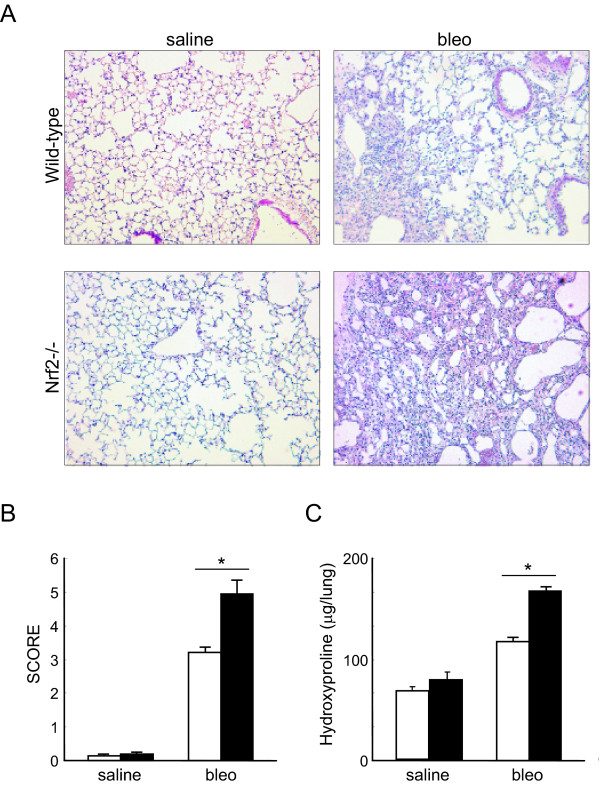
**Bleomycin-induced pulmonary fibrosis is enhanced in *Nrf2-/- *mice**. **(A) **Representative photographs of lungs from WT mice and *Nrf2-/- *mice 28 days after treatment with bleomycin (bleo) or saline. Magnification: ×40. **(B-C) **The score of fibrosis **(B) **and lung hydroxyproline contents **(C) **of WT mice (open bars) and *Nrf2-/- *mice (solid bars) 28 days after treatment with bleomycin (bleo) or saline. n = 6 in each group.*Significant difference (*p*<0.05) between WT mice and *Nrf2-/- *mice.

We then assessed the development of bleomycin-induced pulmonary fibrosis in both WT mice and *Nrf2-/- *mice. Masson's trichrome stain revealed mild thickening of alveolar septa and collagen deposition 28 days after bleomycin administration in the lungs of WT mice (Figure 3A, wild-type, bleo). The degree of pulmonary fibrosis was much greater in *Nrf2-/- *mice, compared with wild-type mice. Dense fibrosis with prominent collagen deposition was observed 28 days after bleomycin administration in the lungs of *Nrf2-/- *mice (Figure 3A, Nrf2-/-, bleo). No abnormal alveolar architecture was observed in the lungs of the saline-administered control group in either WT or *Nrf2-/- *mice (Figures 3A, saline).

We next assessed the degree of pulmonary fibrosis using a scoring method. In both genotypes of mice, the scores of fibrotic lesions were significantly increased 28 days after bleomycin administration, compared with those in saline-administered controls (Figure 3B). However, the scores were significantly higher in *Nrf2-/- *mice than in WT mice at that time (Figure 3B).

We further assessed the degree of pulmonary fibrosis by measuring the lung hydroxyproline content, which we found to be significantly increased 28 days after bleomycin administration in both genotypes (Figure 3C). The concentration, however, was significantly higher in *Nrf2-/- *mice than in WT mice at that time (Figure 3C). These findings suggest that mice lacking Nrf2 are more susceptible to bleomycin-induced pulmonary fibrosis.

### Inflammatory protein expression is enhanced in the lungs of Nrf2-/-mice

**Figure 4:**
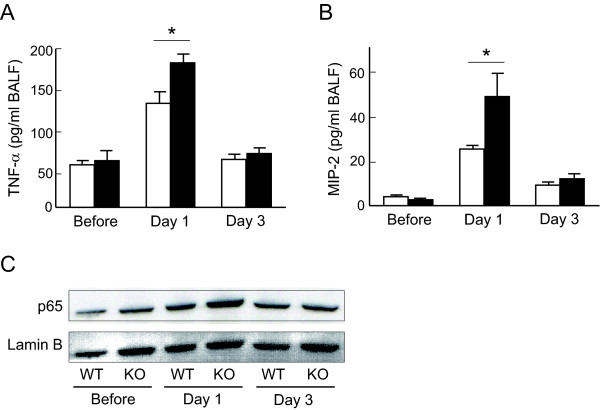
**Inflammatory protein level and activation of NF-κB are elevated in the lungs of *Nrf2-/- *mice**. The concentration of TNF-α **(A) **and MIP-2 **(B) **in bronchoalveolar lavage fluid of WT mice (open bars) or *Nrf2-/- *mice (solid bars) before, and 1 day (Day 1) and 3 days (Day 3) after bleomycin administration. n = 4 in each group. *Significant difference (p < 0.05) between WT mice and *Nrf2-/- *mice. **(C) **Nuclear localization of NF-κB (p65) in the lungs of WT mice or *Nrf2-/- *mice (KO) before, and 1 day (Day 1) and 3 days (Day 3) after bleomycin administration. Lamin B was used as an internal control.

Since neutrophilic pulmonary inflammation was enhanced in the lungs of *Nrf2-/- *mice at 1 and 3 days after bleomycin administration, the levels of TNF-α and MIP-2 were evaluated in the BAL fluid of both genotypes of mice at those time points. The concentration of TNF-α was significantly elevated in the BAL fluid of *Nrf2-/- *mice, compared with WT mice, 1 day after bleomycin administration (Figure 4A). The concentration of MIP-2 was also significantly higher in the BAL fluid of *Nrf2-/- *mice than in that of WT mice 1 day after bleomycin administration (Figure 4B). These findings suggest that the pulmonary levels of inflammatory cytokines and chemokines were elevated in mice lacking Nrf2 by stimulation with bleomycin.

NF-κB is known as a transcription factor that regulates the expression of inflammatory cytokines such as TNF-α and MIP-2. We therefore assessed the activation of NF-κB in the lungs of both WT mice and *Nrf2-/- *mice. The nuclear location of NF-κB was elevated in the lungs of both genotypes 1 day after bleomycin administration. However, the degree of NF-κB nuclear location was much higher in the lungs of *Nrf2-/- *mice than those of WT mice 1 day after bleomycin administration (Figure 4C). These findings indicate that activation of NF-κB is enhanced in the lungs of mice lacking Nrf2.

### Induction of antioxidant enzymes is attenuated in Nrf2-/-mice

**Figure 5:**
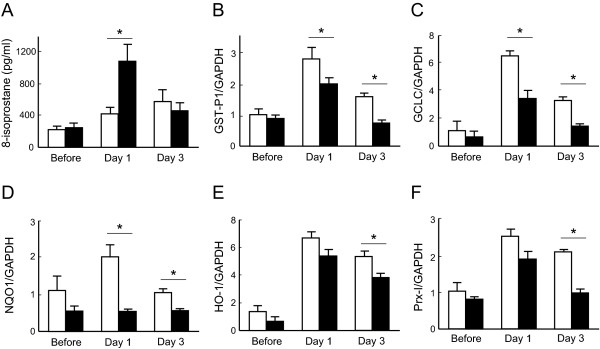
**Induction of antioxidant genes is attenuated in the lungs of *Nrf2-/- *mice**. **(A) **The concentration of 8-isoprostane, an oxidative stress marker, in the lungs of WT mice (open bars) or *Nrf2-/- *mice (solid bars) before, and 1 day and 3 days after bleomycin administration. n = 4 in each group. **(B-F) **The expression of GST-P1 **(B)**, GCLC **(C)**, NQO1 **(D)**, HO-1 **(E)**, and PrxI**(F) **mRNAs in the lungs of WT mice (open bars) or *Nrf2-/- *mice (solid bars) before, and 1 day (Day 1) and 3 days (Day 3) after bleomycin administration. GAPDH was used as an internal control. n = 3 in each group. *Significant difference (p < 0.05) between WT mice and *Nrf2-/- *mice.

Oxidative stress may play an important role in the development of bleomycin-induced pulmonary fibrosis. We therefore assessed the degree of oxidative stress in the lungs of both WT mice and *Nrf2-/- *mice. The concentration of 8-isoprostane, a marker of oxidative stress, was increased significantly in the lung homogenates of *Nrf2-/- *mice 1 day after bleomycin administration, compared with that of WT mice (Figure 5A).

We next assessed the expression of antioxidant and glutathione-related enzyme genes, such as GST-P1, GCLC, NQO1, HO-1, and Prx-I, in the lungs of both WT mice and *Nrf2-/- *mice. Messenger RNA expression of all of these enzymes was significantly induced in the lungs of WT mice by stimulation with bleomycin. However, the expression levels of GST-P1, GCLC, and NQO1 mRNAs were significantly lower in the lungs of *Nrf2-/- *mice than in those of WT mice at both 1 and 3 days after bleomycin administration (Figures 5B-5D). The expression levels of HO-1 and Prx-I mRNAs were also significantly lower in the lungs of *Nrf2-/- *mice than in those of WT mice 3 days after bleomycin administration (Figures 5E and 5F). These findings indicate that oxidative stress is enhanced in the lungs of mice lacking Nrf2 due to the lower induction of antioxidants in response to bleomycin.

### Th2-bias occurs in the lungs of Nrf2-/- mice after bleomycintreatment

**Figure 6:**
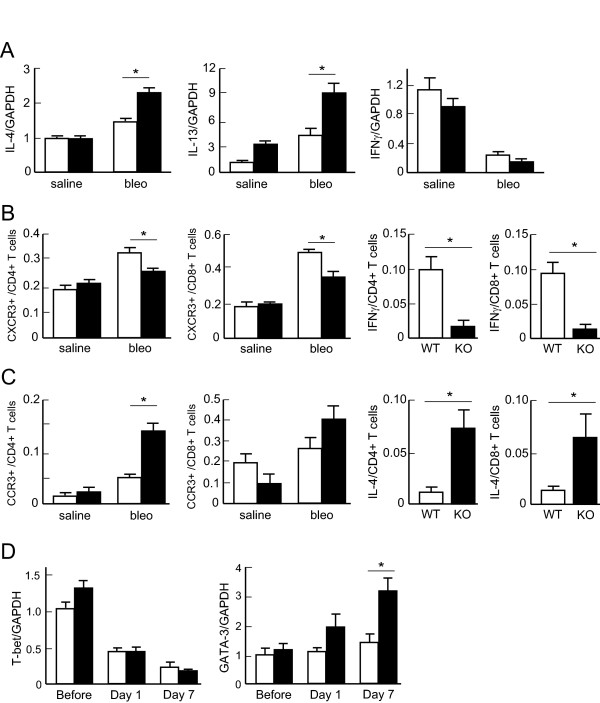
**Lung Th1/Th2 balance is shifted toward Th2 in *Nrf2-/- *mice**. **(A) **The expression of IL-4, IL-13, and IFN-γ in the lungs of WT mice (open bars) or *Nrf2-/- *mice (solid bars) 7 days after treatment with saline or bleomycin (bleo). GAPDH was used as an internal control. n = 6 in each group. **(B-C) **The left panels show the proportion of CXCR3-positive **(B) **or CCR3-positive **(C) **cells among CD4-positive cells or among CD8-positive cells obtained from WT mice (open bars) or *Nrf2-/- *mice (solid bars) 7 days after treatment with saline or bleomycin (bleo). n = 6 in each group. The right panels show the proportion of IFN-γ-producing **(B) **or IL-4-producing **(C) **cells among CD4-positive cells or among CD8-positive cells obtained from WT mice or *Nrf2-/- *mice (KO) 7 days after treatment with bleomycin. n = 6 in each group. **(D) **The expression of T-bet and GATA-3 in the lymphocytes obtained from the lungs of WT mice (open bars) or *Nrf2-/- *mice (solid bars) 7 days after treatment with saline or bleomycin (bleo). GAPDH was used as an internal control. n = 6 in each group. *Significantly different (p < 0.05) between WT mice and *Nrf2-/- *mice.

Th2 cytokines play an important role in the pathogenesis of pulmonary fibrosis. We therefore assessed Th1/Th2 cytokine levels in the lungs of both WT mice and *Nrf2-/- *mice after bleomycin administration. The mRNA expression of the Th2 cytokines IL-4 and IL-13 was increased in the lungs of both genotypes after bleomycin administration. However, the expression of both IL-4 and IL-13 mRNAs was significantly higher in the lungs of *Nrf2-/- *mice than in those of WT mice 7 days after bleomycin treatment (Figure 6A, left and center panels). Although the expression of IFN-γ, a Th1 cytokine, was decreased significantly in the lungs of both genotypes of mice after bleomycin administration, the level was not different between WT mice and *Nrf2-/- *mice (Figure 6A, right panel).

We then assessed the appearance of Th1 and Th2 cells in the lungs of both WT mice and *Nrf2-/- *mice using their cell surface markers CXCR3 and CCR3, respectively. The proportion of CXCR3-positive cells among CD4+ T cells was significantly increased in the lungs of WT mice but not in those of *Nrf2-/- *mice 7 days after bleomycin administration, compared with the corresponding saline-administered control. Between the genotypes, the proportion was significantly lower in *Nrf2-/- *mice than in WT mice at that time point (Figure 6B, left panel). Similarly, the proportion of CXCR3-positive cells among CD8+ T cells was significantly lower in *Nrf2-/- *mice than in WT mice 7 days after bleomycin administration (Figure 6B, middle-left panel). We also assessed Th1 and Th2 cytokine production in lung T cells. The proportion of IFN-γ-producing cells among CD4+ T cells was significantly lower in *Nrf2-/- *mice than in WT mice 7 days after bleomycin administration (Figure 6B, middle-right panel). The proportion of IFN-γ-producing cells among CD8+ T cells was significantly lower in *Nrf2-/- *mice than in WT mice at that time point (Figure 6B, right panel).

The proportion of CCR3-positive cells among CD4+ T cells increased markedly in the lungs of *Nrf2-/- *mice but not in those of WT mice 7 days after bleomycin administration, compared with the saline-administered control. Between genotypes, the proportion was significantly higher in *Nrf2-/- *mice than in WT mice 7 days after bleomycin administration (Figure 6C, left panel). Although the proportion of CCR3-positive cells among CD8+ T cells increased in the lungs of *Nrf2-/- *mice 7 days after bleomycin administration, compared with the saline-administered control, the proportion was not different between the mouse genotypes after bleomycin administration (Figure 6C, middle-left panel). The proportion of IL-4-producing cells among CD4+ T cells was significantly higher in *Nrf2-/- *mice than in WT mice 7 days after bleomycin administration (Figure 6C, middle-right panel). The proportion of IL-4-producing cells among CD8+ T cells was also significantly higher in *Nrf2-/- *mice than in WT mice at that time point (Figure 6C, right panel).

The transcription factors T-bet and GATA3 are known as the master regulators of Th1 and Th2 differentiation, respectively. We therefore assessed the expression of both T-bet and GATA3 in the lymphocytes obtained from the lungs of WT mice and *Nrf2-/- *mice. The expression of T-bet mRNA was decreased by 7 days after bleomycin administration in both genotypes of mice (Figure 6D, left panel). However, the expression of T-bet was not different between WT mice and *Nrf2-/- *mice (Figure 6C, left panel). The expression of GATA3 mRNA was elevated in the lungs of *Nrf2-/- *mice after bleomycin treatment, while the expression was not upregulated in the lungs of WT mice after bleomycin administration (Figure 6D, right panel). The expression of GATA3 was significantly higher in the lungs of *Nrf2-/- *mice than in those of WT mice 7 days after bleomycin treatment (Figure 6D, right panel).

## Discussion

In this study, we demonstrated that mice lacking Nrf2 are highly susceptible to bleomycin-induced pulmonary inflammation and fibrosis. Bleomycin is a chemotherapeutic drug used for treatment of various human carcinomas. It has been reported that administration of a high-dose of bleomycin often induces acute lung injury and pulmonary fibrosis in both human and experimental animals [25,26]. Bleomycin can directly produce ROS during the process of reaction with DNA [27]. Moreover, bleomycin indirectly increases ROS by activating inflammatory cells accumulated in the lung to generate ROS [28,29]. In the present study, the concentration of 8-isoprostane, a marker of oxidative stress, was significantly higher in the lungs of *Nrf2-/- *mice than in those of WT mice after bleomycin administration. These results indicate that oxidative stress is enhanced in the lungs of *Nrf2-/- *mice after bleomycin administration. Thus, Nrf2 may be an important host factor to protect against oxidant-induced tissue damages.

Nrf2-mediated defense mechanisms against oxidative stress have recently been elucidated at the molecular level. Under unstimulated conditions, Nrf2 is retained in the cytoplasm through binding to Kelch-like ECH-associated protein-1 (Keap1) and is maintained at a reduced level by the Keap1-dependent ubiquitination and proteosomal degradation systems[30,31]. Upon exposure to oxidative stress or xenobiotic stress, Keap1-dependent ubiquitin ligase activity is inhibited and Nrf2 can translocate to the nucleus, where it forms a heterodimer with small Maf proteins and binds to ARE. Pulmonary Nrf2 effecter genes bearing AREs include a majority of phase II detoxifying enzymes such as GST isozymes, GCLC, and NQO1, as well as stress proteins such as HO-1, Prx I, thioredoxin (Trx), and Trx reductase [32]. In the present study, the induction of these cytoprotective genes following exposure to bleomycin was consistently and markedly attenuated in the lungs of *Nrf2-/- *mice relative to that in the lungs of WT mice. Thus, the reduced induction of ARE-mediated antioxidant defense enzymes in the lungs of *Nrf2-/- *mice suggests that these enzymes contribute to Nrf2-mediated protection against bleomycin-induced pulmonary inflammation and fibrosis. The protective role of ARE-mediated antioxidant enzymes in experimental fibrosis has been demonstrated. Activation of Nrf2 by a polyphenol epigallocatechin-3-gallate treatment reduced bleomycin-induced lung injury and inflammation in rats by inducing phase II enzymes such as GST and NQO1 [33]. The pulmonary fibrosis and inflammation induced by bleomycin were prevented in mice overexpressing thioredoxin [34].

Although the pathogenesis of pulmonary fibrosis is not fully understood, a newer hypothesis suggests that pulmonary fibrosis is the culmination of the wound-healing responses to sequential acute lung injury [3]. The fibrotic response itself is modified by the predominant inflammatory phenotype, either Th1 or Th2. Although the Th2 and Th1 phenotypes are not as well defined in idiopathic pulmonary fibrosis as they are in asthma and animal models, their potential importance is one rationale for trials in which immunomodulators such as IFN-γ are used in an attempt to switch the inflammatory responses to a more Th1-like phenotype. In the present study, we found that the levels of Th2 cytokines IL-4 and IL-13 were elevated in the lungs of *Nrf2-/- *mice after bleomycin administration. These cytokines enhance the fibrotic process by augmenting fibroblast proliferation and collagen production, and are required for the initiation and maintenance of pulmonary fibrosis [35-37]. Both IL-4 and IL-13 are mainly produced and secreted by Th2 cells. Consistently, the present study showed that the proportion of CCR3-positive Th2 cells was significantly increased while the proportion of CXCR4-positive Th1 cells was decreased in the lungs of *Nrf2-/- *mice after bleomycin administration. The measurement of intracellular cytokine levels also revealed that the proportion of IL-4-producing T cells was increased whereas the proportion of IFN-γ-producing T cells was decreased in the lungs of *Nrf2-/- *mice after bleomycin administration. These findings suggest that the Th1/Th2 balance is shifted toward Th2 in the lungs of *Nrf2-/- *mice by exposure to bleomycin. Nrf2 may affect the differentiation and cytokine production of T cells rather than baseline homing of Th1/Th2 cells in the lungs, since the level of Th1 and Th2 cells was not different between the lungs of WT mice and *Nrf2-/- *mice under the unstimulated condition.

The differentiation of Th1/Th2 cells is regulated by the transcription factors T-bet and GATA3, respectively. GATA3, a member of the GATA family of zinc-finger transcription factors, is known to be a key regulator of Th2 development. It has been demonstrated that antisense GATA3 inhibits the expression of all Th2 cytokine genes in the Th2 clone D10 [38]. In transgenic mice, elevated GATA3 in CD4+ T cells induces Th2 cytokine gene expression in developing Th1 cells [38]. It has also been reported that GATA3 regulates the locus accessibility of the IL-4 and IL-13 genes with chromatin remodeling [39,40]. These findings suggest that GATA3 allows the expression of Th2 cytokines by functioning as a transcription factor as well as by modifying the chromatin structure of these cytokines. Our previous study demonstrated that the development of bleomycin-induced pulmonary fibrosis was significantly enhanced in mice overexpressing GATA3 with a lung Th2 cytokine bias [41]. In the present study, expression of GATA3 was elevated significantly in the lymphocytes obtained from the lungs of *Nrf2-/- *mice, whereas the GATA3 level was not altered in the lymphocytes from WT mice after bleomycin administration. Thus, GATA3-mediated Th2 cell differentiation and Th2 cytokine induction are an additional mechanism for aggravation of bleomycin-induced pulmonary fibrosis in mice lacking Nrf2. The relationship between Nrf2 and Th2 bias has been reported previously. Genetic deletion of Nrf2 renders mice more susceptible to Th2-driven allergic airway inflammation [42]. Furthermore, stimulation of *Nrf2-/- *dendritic cells with ambient particulate matter augmented oxidative stress and Th2 cytokine production as compared with *Nrf2 *wild-type dendritic cells [19].

Although it is unclear why Th2 bias occurs in *Nrf2-/- *mice after bleomycin exposure, it has been demonstrated that oxidative stress favors a Th2-polarizing condition. King et al. have demonstrated that exposure of T cells to 2,3-dimethoxy-1,4-naphthoquinone, which generates a low level of superoxide anion, resulted in the growth of cells expressing CCR4, and a decrease in cells expressing CXCR3, indicating phenotypic conversion to Th2 cells by activation of signal transducer and activator of transcription 6 (STAT6), leading to the induction of GATA3 [43]. In the present model, we found that phosphorylated STAT6 levels were enhanced in the lung lymphocytes of *Nrf2-/- *mice after bleomycin exposure (data not shown). These findings suggest that enhanced oxidative stress promotes Th2 cell differentiation and Th2 cytokine production by activating STAT6, followed by activation of GATA3. Thus, Nrf2 may be a critical regulator for Th1/Th2 balance in the lungs exposed to oxidants or electrophiles.

In the present study, the activation of NF-κB was enhanced in the lungs of *Nrf2-/- *mice after bleomycin administration. NF-κB plays a cardinal role in the development of pulmonary inflammation through transcriptional activation of many proinflammatory genes, including the genes of cytokines and chemokines [44]. In the present study, the levels of TNF-α and MIP-2 were also consistently enhanced in *Nrf2-/- *mice. NF-κB is known as a redox-sensitive transcription factor that is activated by ROS [45,46]. It is therefore likely that Nrf2 inhibits the activation of NF-κB by reducing cellular oxidant levels. Lung macrophages are thought to be the primary source of those cytokines induced by NF-κB. We have demonstrated that Nrf2-mediated transcription of cytoprotective genes is mainly activated in macrophages in response to environmental stimuli [47,48]. It is therefore reasonable to hypothesize that the aberrant gene expression in *Nrf2-/- *macrophages may cause an overwhelming inflammatory response and thus evoke pulmonary fibrosis.

## Conclusions

The present study showed that Nrf2 protects against the development of bleomycin-induced pulmonary inflammation and fibrosis by regulating the cellular redox level and Th1/Th2 balance. Nrf2 has an advantage over a single antioxidant molecule for the treatment of acute lung injury and pulmonary fibrosis, since Nrf2 coordinately induces a variety of self-defense genes, including antioxidant and phase II enzymes. In addition, Nrf2 is expressed abundantly in macrophages, cells which are easily collected by BAL. Acute respiratory distress syndrome and idiopathic pulmonary fibrosis are lethal disorders for which effective therapeutic approaches are not readily available. Although transcription factor regulation therapy cannot currently be used for the treatment of these diseases, we believe that the present results may lead to new therapeutic options.

## Abbreviations

Nrf2: NF-E2 related factor-2; Keap1: Kelch-like ECH-associated protein-1; ROS: reactive oxygen species; GST: glutathione-S-transferase; NQO1: NADP(H): quinine oxidoreductase; GCLC: glutamate-cysteine ligase catalytic; HO-1: heme oxygenase-1; Prx-I: peroxiredoxin I; ARE: antioxidant response element; TNF-α: tumor necrosis factor-α; MIP-2: macrophage inflammatory protein-2; STAT6: signal transducer and activator of transcription 6; GATA3: GATA binding protein-3; NF-κB: nuclear factor-kappaB.

## Competing interests

The authors declare that they have no competing interests.

## Authors' contributions

NK performed the *in vitro *and *in vivo *experiments and drafted the initial version of the manuscript. YM carried out flow cytometry. YY and NH contributed to the *in vivo *and *in vitro *experiments. KI and MY generated the knockout mouse. YI and NH participated in the design and coordination of the study and drafted the final manuscript. All authors have read and approved the final manuscript.
